# Data-driven model as a post-process for daily streamflow prediction in ungauged basins

**DOI:** 10.1016/j.heliyon.2025.e42512

**Published:** 2025-02-06

**Authors:** Jeonghyeon Choi, Sangdan Kim

**Affiliations:** a Forecast and Contral Division, Nakdong River Flood Control Office, Ministry of Environment, 1233-88, Nakdongnam-ro, Saha-gu, Busan, 49300, Republic of Korea; b Division of Earth Environmental System Science (Major in Environmental Engineering), Pukyong National University, 45 Yongso-ro, Nam-gu, Busan, 48513, Republic of Korea

**Keywords:** Data-driven model, Hybrid model, Post-process, Process-based model, Streamflow prediction in ungauged basins

## Abstract

Streamflow prediction in ungauged basins (PUB) remains a significant challenge in water resource planning and management. Although recent studies have proposed various approaches to reduce prediction errors using data-driven models (DDMs), further efforts are needed to improve applicability and accuracy of predictions in ungauged basins. This study proposes a framework that utilizes DDM as a post-processor to enhance the PUB performance of process-based models (PBMs) or DDMs and investigates its applicability. For this purpose, the Parsimonious EcoHydrologic Model (PEHM) was selected as a PBM, and Long Short-Term Memory (LSTM) and Random Forest (RF) were chosen as DDMs. We tested the proposed approach on 28 basins in Korea, which were assumed to be ungauged. First, PEHM and LSTM were used separately to predict streamflow in ungauged basins. Subsequently, RF was employed as the main DDM for post-processing, and the post-processing effects of LSTM were also examined. The results in this study demonstrate the potential value of various post-processing approaches in improving streamflow prediction in ungauged basins.

## Graphical abstract

**Figure:**
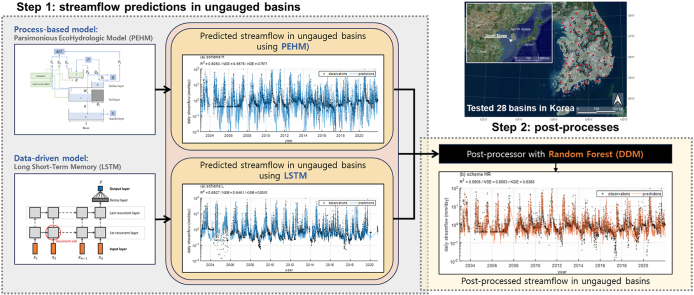

## 1 Introduction

Streamflow prediction in ungauged basins (PUB) remains a significant challenge in water resource planning and management [1,2]. Process-based models (PBMs), which can take into account the complex interactions between climate and geographical factors such as topography, land cover, and geology, are one of the best alternatives for predicting streamflow in ungauged basins [3]. PUB involves "donating" calibrated parameters of a PBM from a gauged basin to an ungauged basin of interest [2,[4], [5], [6], [7], [8], [9], [10]]. The performance of regionalization approaches using information from gauged basins selected based on hydrologic similarity, spatial proximity, or various other indicators with ungauged basins has been demonstrated in previous studies [1,11,12]. However, as the regionalization distance increases, performance gradually deteriorates [10], and the regionalization approach may not be suitable in areas where streamflow gauges are very limited or sparsely distributed. Therefore, data scarcity and regionalization issues are particularly pronounced in high-altitude and complex terrain regions with few streamflow gauges [13]. Additionally, the impact of the user's judgment in selecting donor basins cannot be ignored [14]. In recent years, various studies have proposed using satellite data instead of observed streamflow data for the calibration in ungauged basins [3,[13], [14], [15], [16], [17]]. However, PBMs are limited by uncertainties in their datasets, heterogeneity in their parameters, scale effects, and nonlinearities in their process dynamics [[18], [19], [20]], and unsuitable model structures and inappropriate calibration procedures also negatively impact the performance of PUBs [21].

Recently, there has been growing interest in the hydrology field in improving the performance of streamflow prediction using data-driven models (DDMs) [[22], [23], [24]]. This is achieved either by directly replacing PBMs [25,26] or by using them in conjunction [27,28]. DDMs do not need to model the complex relationships between input and output variables or to explicitly consider the governing physical laws [29]. Although they require a large amount of data for training, they do not explicitly demand specific data types (e.g., hard-to-obtain soil data). A particular type of deep learning model that has been gaining popularity in hydrology over the past few years is the Long Short-Term Memory (LSTM) network, which possesses the ability to handle sequential data and time series [[30], [31], [32]]. Kratzert et al. [33] aimed to predict streamflow in ungauged basins using only meteorological and basin characteristics. They demonstrated that their LSTM model could provide better PUB estimates than a well-known PBM, calibrated using observed streamflow. Similarly, Choi et al. [34] reported satisfactory results in the PUB performance of an LSTM trained with well-observed basins in Korea, marking a stark contrast to parameter transfer using PBMs. When applied to ungauged basins, a PBM calibrated on a specific dataset will inevitably experience performance degradation [35]. In contrast, Ayzel et al. [36] argued that DDMs are highly efficient in extracting information from large datasets across an entire region, and Kratzert et al. [37] mentioned that this could serve as an added value of data-driven approaches. However, other studies have reported difficulties in using LSTMs for predicting streamflow under extreme conditions and in basins with complex groundwater-river interactions or significant human interventions [38,39]. In general, this may be due to the use of limited training datasets that do not include extreme conditions and a lack of representativeness in the training data for capturing the relationships between input and output data.

Recent studies have explored the potential of machine learning techniques as a means to overcome the limitations of using either PBM or DDM alone [40]. Xu et al. [41] demonstrated that machine learning-based DDMs could improve performance by correcting errors in outputs predicted by PBMs. Konapala et al. [42] also validated the potential of a hybrid model that combines the Sacramento soil moisture accounting model with LSTM for predicting streamflow. Won et al. [26] further enhanced the performance of PUB by correcting errors in outputs predicted by LSTM using another machine learning technique, Random Forest (RF).

The main goal of this study is to investigate the applicability of DDM as a post-processor to reduce prediction errors of PBMs or DDMs for streamflow prediction in ungauged basins. Therefore, a framework using DDMs as a post-processor is proposed. DDMs as post-processing processors are employed to correct errors in model outputs generated by preceding models. While there have been attempts to use DDMs to correct errors in streamflow predictions generated by PBMs, research on how much this approach can improve the performance of PUB is scarce. Furthermore, the application of DDMs as a post-processing process to simultaneously consider and correct errors in outputs from both PBMs and DDMs remains a research gap.

## 2 Material and methodology

### 2.1 Data sources

**Fig. 1:**
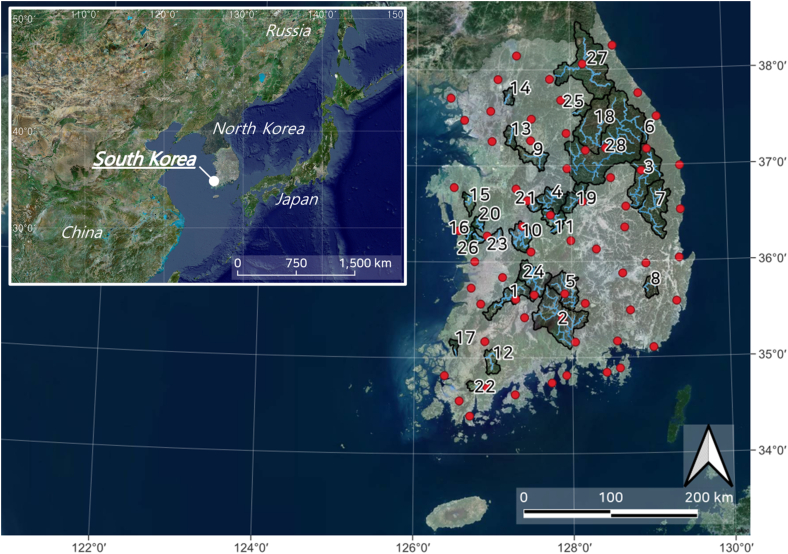
Location of study basins (red circles: meteorological stations).

In this study, daily streamflow observation data (2001–2021) from 28 basins located in South Korea were utilized. This data is provided by the Ministry of Environment's Water Resources Management Information System (WAMIS; wamis.go.kr). The locations of the basins and the observation stations are presented in Fig. 1, and the hydrological characteristics of each basin are shown in Table 1.

**Table 1:** Hydrologic characteristics of the selected 28 basins.

Basin ID	Area (km^2^)	Mean annual rainfall (mm)	Mean annual ETo (mm)	Mean annual runoff (mm)	Mean CN	Mean Impervious rate
1	763.5	1384.5	1007.2	751.8	69.6	0.0750
2	2281.7	1504.4	1070.4	930.1	65.2	0.0582
3	1590.7	1174.8	993.1	531.5	61.5	0.0579
4	676.7	1285.7	1013.3	612.3	68.7	0.0464
5	928.9	1317.6	1060.3	651.1	59.5	0.0629
6	120.7	1306.7	949.0	649.4	70.1	0.0362
7	1367.7	1117.8	1113.4	406.8	67.8	0.0509
8	301.9	1150.0	1127.2	554.1	68.6	0.0516
9	519.5	1278.5	1009.0	558.0	65.0	0.0807
10	609.1	1309.6	1029.1	700.7	65.6	0.1312
11	491.2	1303.9	996.2	548.3	65.2	0.0564
12	411.0	1446.9	1070.1	501.8	64.5	0.0646
13	262.4	1304.8	1011.8	756.5	63.2	0.1314
14	201.5	1391.7	1059.1	443.4	63.9	0.0878
15	221.4	1219.5	969.2	548.9	72.8	0.0919
16	162.3	1158.1	933.4	469.8	59.1	0.0729
17	114.6	1320.7	1041.8	515.1	74.1	0.0879
18	1615.6	1296.6	1023.7	558.3	61.4	0.0418
19	612.4	1310.0	1065.9	708.3	63.7	0.0473
20	208.6	1272.4	992.2	577.6	63.9	0.0571
21	167.6	1221.7	1051.3	656.0	69.7	0.1220
22	152.5	1490.3	1048.4	759.6	67.2	0.0706
23	132.0	1293.0	1002.7	520.0	72.7	0.0732
24	930.4	1441.3	1002.4	824.2	64.3	0.0784
25	207.9	1317.6	1001.7	569.3	54.1	0.0458
26	155.9	1283.0	997.4	435.6	70.1	0.0768
27	2694.3	1262.2	1025.3	755.4	53.8	0.0586
28	6661.5	1300.4	1038.8	596.0	64.2	0.0491

For reference, it should be noted that Basin 18 overlaps with Basin 28.

The daily precipitation and meteorological data for the selected 28 basins were collected from the Meteorological Data Portal (data.kma.go.kr), Korea Meteorological Administration (KMA). The meteorological data includes minimum and maximum air temperatures, average wind speed, and relative humidity. Daily reference evapotranspiration (ETo) was calculated using the Penman-Monteith method proposed by Allen et al. [43] based on the collected meteorological data. Hydrogeomorphical information for each basin, such as Curve Number (CN), saturated hydraulic conductivity (Ks), and imperviousness ratio (Ri), was extracted based on soil and land cover maps provided by the Ministry of Environment and the Rural Development Administration.

The satellite data used for calibrating the parameters of the PBM in this study is the Leaf Area Index (LAI), which is the Level-4 MODIS global LAI product (MOD15A2H) provided by the Land Processes Distributed Active Archive Centre (LP DAAC). This data has a spatial resolution of 500 m and a temporal resolution of 8 days.

By applying area-weighted averaging to pixels across the catchment boundaries, pixel values from satellite images were assigned to the study basins, creating an input data set corresponding to observed streamflow data. This method is commonly employed in processing satellite observation data for hydrological modeling [44,45]. The spatial averages of precipitation and ETo were calculated using the Thiessen polygon technique. Ultimately, spatially averaged data for precipitation, ETo, and LAI were constructed.

### 2.2 Process-based model

**Fig. 2:**
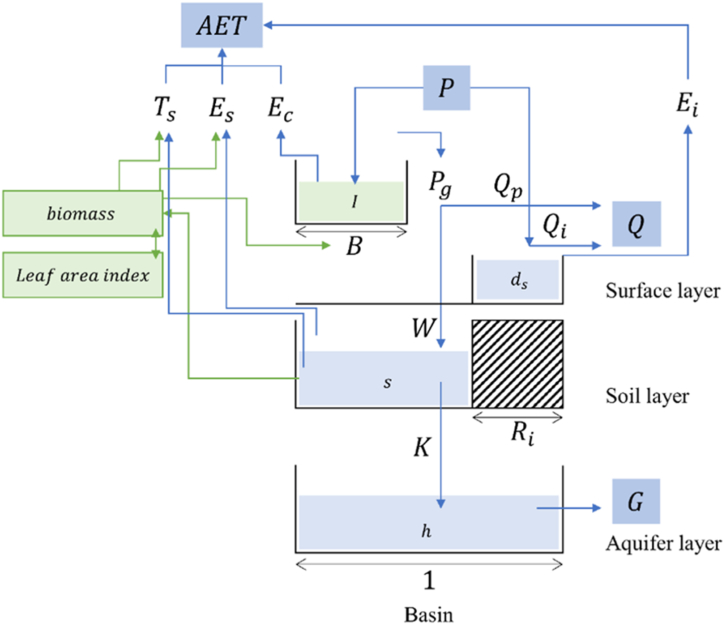
Schematic diagram of the Parsimonious EcoHydrologic Model (PEHM). Reproduced with permission from Choi et al. [3], Science of the Total Environment, 903, 166617, 2023, Elsevier. License No. 5960721295435.

Our fundamental goal is to explore ways to improve the PUB performance of a PBM or a DDM. In an ungauged basin, the absence of observed data (primarily streamflow) makes it impossible to calibrate parameters for the basin. As one of several solutions to this problem, Choi et al. [3] proposed using satellite data directly for model calibration. They employed the Parsimonious EcoHydrologic Model (PEHM), a PBM with a vegetation module. Drawing on the concept of the Tank model [46], this model is composed of three layers: surface, soil, and aquifer layers, and calculates the Leaf Area Index (LAI) over time, which interacts with various hydrological elements simulated in each layer (see Fig. 2).

By calibrating the parameters of the PEHM with LAI data, they could satisfactorily predict streamflow in ungauged basins in Korea. This study adopted the PEHM, whose applicability to PUB has been demonstrated, as the PBM. More detailed information about this model can be found in the study by Choi et al. [3].

### 2.3 Data-driven models

This study chose the LSTM network as the preceding DDM for streamflow prediction. Furthermore, RF was utilized in the post-processing step to correct errors in the model outputs.

#### 2.3.1 Long Short-Term Memory for streamflow prediction

LSTM is a type of Recurrent Neural Network (RNN) known for its exceptional ability to process sequential data and time series, making it a consistently popular deep-learning model in the field of hydrology [30,31]. LSTM comprises memory cells, neurons with self-recurrent connections, and three nonlinear gates (forget, input, and output gates) that control the flow of information into and out of the cell. The forget gate decides which information to retain or discard from the previous time step, allowing LSTM to learn long-term dependencies that other RNN models struggle with. The input gate regulates which information should be added to update the cell state, while the output gate determines how much of the cell state should be used to generate the output. Consequently, the structure of LSTM addresses the issue of exploding or vanishing gradients that traditional RNN structures face, offering an advantage in learning long-term dependencies from more extended memory [47]. Due to these characteristics, LSTM is particularly well-suited for daily simulations involving delays spanning several years between precipitation and streamflow [48].

#### 2.3.2 Random Forest for post-processing

RF was used to correct errors in streamflow predictions generated by preceding models (PEHM and LSTM). RF is a type of ensemble decision tree introduced by Breiman [49], which reduces the model's prediction variance through random sampling while maintaining robust performance even in noisy input variables [50,51]. RF learns from random subsets of the dataset and generates the final prediction by taking the average of predictions made by all ensemble members. This approach helps capture the diverse characteristics of the data and prevents overfitting. More detailed information about RF can be found in Hastie et al. [52].

### 2.4 Experimental settings

**Fig. 3:**
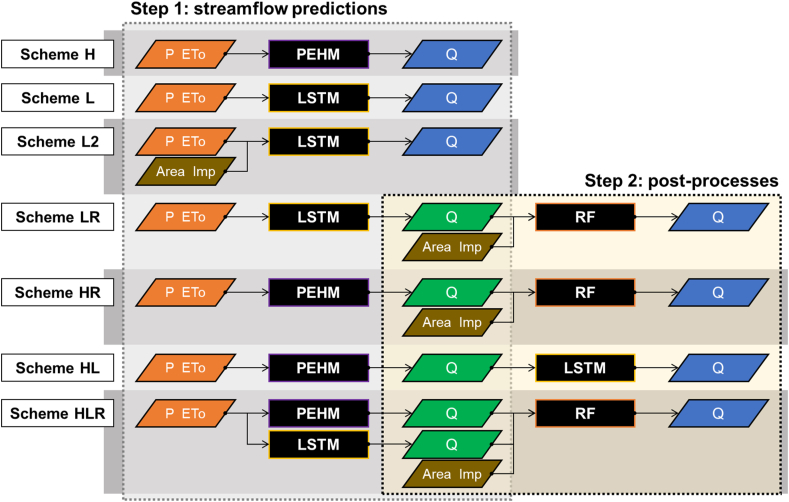
The process flow diagram for each scheme. In the diagram, P and PET represent meteorological input data, namely precipitation and reference evapotranspiration, respectively. Area and Imp represent the basin area and impervious area ratio, respectively, and are used as additional training data in some schemes. Q represents the streamflow predicted by each model, where in step 2, the streamflow generated in step 1 is used as input data for the post-processing model in step 2 to produce the final streamflow.

The experimental setup of this study was designed to examine the applicability of the post-processing processor to enhance the PUB performance of the selected preceding models. To this end, various strategies were established as follows (see Fig. 3).

(1) Predicting streamflow in ungauged basins using PEHM (scheme H);
(2) Predicting streamflow in ungauged basins using LSTM (with precipitation and ETo as inputs, scheme L);
(3) Predicting streamflow in ungauged basins using LSTM (with precipitation, ETo, and topographical information as inputs, scheme L2);
(4) Post-processing the output (streamflow) of scheme L using RF (scheme LR);
(5) Post-processing the output of scheme H using RF (scheme HR);
(6) Post-processing the output of scheme H using LSTM (scheme HL); and
(7) Post-processing the outputs of schemes H and L together using RF (scheme HLR).

The first step is to use PEHM and LSTM alone to predict streamflow in ungauged basins. Before performing predictions, it is essential to calibrate and train the models properly. To construct the PBM (PEHM) for the ungauged basins, hydrogeomorphical information for the 28 basins was inputted, and the parameters were calibrated for each basin using MODIS LAI data (2016–2020). Subsequently, daily streamflows from 2001 to 2021 were predicted using the 28 PEHMs constructed for the ungauged basins.

Unlike PEHM, predicting streamflow with LSTM requires direct access to streamflow data as target data. However, since this study aims to predict streamflow in ungauged basins, a strategy was adopted to train a single LSTM using the integrated data from all available gauged basins. This approach can be seen as an extension of the traditional regionalization approach, which uses information from an appropriate gauged basin (donor basin) to predict streamflow in an ungauged basin (target basin) into a data-driven model framework [34]. The extensive training dataset plays a crucial role in enabling the model to learn more general and abstract input-output relationships [53], which can help the model better understand the rainfall-runoff process. Therefore, the Leave-one-out cross-validation (LOOCV) method is applied using all 28 basins. For each iteration, one of the 28 basins is assumed to be ungauged, and the model is trained using data from the remaining 27 basins. The trained model is then used to evaluate the streamflow prediction performance for the assumed ungauged basin. This procedure is repeated until each basin has been tested once, resulting in the construction of 28 models and the performance evaluation for the 28 ungauged basins. For basin i, the streamflow data $Q_{t}^{i}$ for day t was set as the target, and a network was constructed using precipitation $P^{i}$, reference evapotranspiration ${ET}_{o}^{i}$, and streamflow data $Q^{i}$ from days t −1 to t −365 as input data (for scheme L2, hydrogeomorphic information of the basin was added). In other words, our LSTM requires 365 days of antecedent meteorological data as input data to compute one streamflow. The daily streamflow (2002–2021) for a given ungauged basin was predicted from the corresponding LSTM for that ungauged basin among the 28 LSTMs constructed using the LOOCV method.

The second step involves post-processing to reduce errors in the streamflow predictions for ungauged basins made by PBM (schemes H) and/or DDM (scheme L). The LOOCV method was also used in the post-processing stage to predict streamflow for ungauged basins by learning from the information of the remaining 27 basins. In other words, when assuming Basin 1 as an ungauged basin, the RF, trained on streamflow predictions made by PEHM and/or LSTM and corresponding observations from Basins 2 to 28, was used to post-process the streamflow predictions for Basin 1 made by PEHM and/or LSTM. This process was repeated for all basins from Basin 1 to Basin 28.

For this purpose, RFs were constructed to learn the relationship between the model outputs $Q_{p,t}^{i}$ (including Hydrogeomorphical information) and the observed streamflow $Q_{t}^{i}$ on day t for the remaining 27 basins, excluding the ungauged basin (see Fig. 3). By inputting the predicted model outputs for the ungauged basin into these RFs, the streamflow was post-processed (schemes LR, HR, and HLR). Post-processing using LSTM instead of RF was also attempted to improve the streamflow predictions from PEHM for the ungauged basins (scheme HL). In this case, the input sequence length was set to 365 days, equal to the first step.

For reference, in the first stage of LSTM modeling, we used a 2-layer network where the cell/hidden state length of each layer was set to 30. To prevent the model from overfitting between layers, we applied a 30 % dropout. Early stopping was applied if there was no improvement in learning after 10 epochs under the condition of validation_split = 0.25. Dropout is a method used to prevent overfitting during the training process by setting the output of some neurons in a layer to 0 [Srivastava et al., 2014]. A batch size of 512 samples was used during model training. We employed Adaptive Moment Estimation (Adam) as the optimizer to find optimal values, and the loss function used was mean squared error (MSE). Adaptive moment estimation, one of the commonly used optimizers, was applied for optimization. All other hyperparameters were set to default values. In the second stage of RF modeling, aside from applying the 5-fold cross-validation technique, all hyperparameters were set to default values.

### 2.5 Performance metrics

Various performance metrics were used to compare the performance of the seven schemes constructed in this study. We evaluated performance using the coefficient of determination (R^2^), the Nash-Sutcliffe efficiency (NSE) [54] and the Kling-Gupta efficiency (KGE) [55] metrics, which are widely used in hydrological studies.

(1) $$R^{2}=1-\frac{\sum_{i=1}^{N}\left(F_{o,i}-\bar{F_{o}}\right)}{\sum_{i=1}^{N}\left(F_{o,i}-F_{s,i}\right)}$$

(2) $$NSE=1-\frac{\sum_{i=1}^{N}\left(F_{s,i}-F_{o,i}\right)}{\sum_{i=1}^{N}{\left(F_{o,i}-\bar{F_{o}}\right)}^{2}}$$

(3) $$KGE=1-\sqrt{{\left(\gamma^{\prime }-1\right)}^{2}+{\left(\alpha^{\prime }-1\right)}^{2}+{\left(\beta^{\prime }-1\right)}^{2}}$$

where N is the number of observations, $F_{s,i}$ is the simulated streamflow, $F_{o,i}$ is the observed streamflow, $\bar{F_{o}}$ is the mean of the observed streamflow, $\gamma^{\prime }$ is the linear correlation coefficient between the observed and simulated data, $\alpha^{\prime }$ is the ratio of the standard deviation of the observed and simulated data, and $\beta^{\prime }$ is the ratio of the mean of the observed data to the mean of the simulated data. For reference, performance metric values of 1 indicate perfect correspondence between predicted and observed values.

Furthermore, to measure the average tendency of predicted values compared to observed values across different flow scales, the percent bias (Pbias) was calculated under four hydrological conditions: overall, high-flow (exceedance probability >0.02), low-flow (exceedance probability <0.3), and medium-flow (the remaining intervals). Pbias <0 indicates an underestimation of predictions compared to observations, while Pbias >0 indicates an overestimation. All metrics were calculated for the results from 2003 to 2021.

## 3 Results and discussion

### 3.1 Summary of prediction performance in ungauged basins

**Fig. 4:**
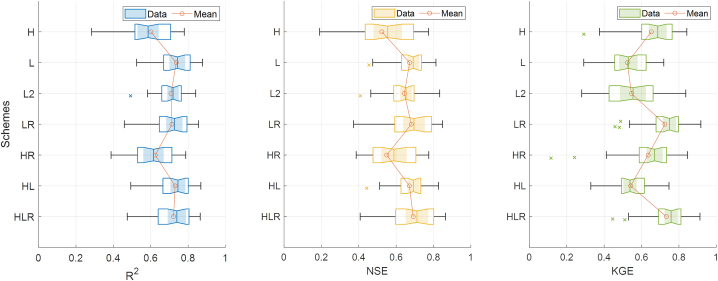
Performance metrics (R^2^, NSE, and KGE) between observations and predictions from each scheme. Each box represents the performance across the 28 ungauged basins, with the x-mark indicating outliers. The circle on the solid lines represents the average performance. Note that the value on the side of a box is its median value, the outliers are values larger than 1.5 times the interquartile range from the 25th or 75th percentile (edge of each box), and the whisks are displayed from the edge of each box to a value that is not considered an outlier.

To predict streamflow in ungauged basins, we utilized the PBM (i.e., PEHM), calibrated with satellite data, and the DDM (i.e., LSTM), trained by integrating information from multiple gauged basins. Subsequently, RF was combined as a post-processor to improve the prediction results. Fig. 4 summarizes the results of the models composed of seven schemes.

While the relative performance varies depending on the performance metric chosen, the average performance of each scheme (the circles on the soil lines in Fig. 4) shows a performance of better than 0.5 regardless of the metric. For reference, the threshold for satisfactory model performance efficiency for NSE and KGE is 0.5 [56,57]. Detailed analysis and discussion of the results are described in subsequent sections.

### 3.2 Streamflow prediction in ungauged basins

In this section, we aimed to (1) compare the streamflow prediction performance of the PBM (scheme H with PEHM) and the DDM (scheme L with LSTM) for predicting streamflow in ungauged basins and (2) compare the advantages and disadvantages of constructing Hybrid models that include a post-processing stage using RF (schemes HR and LR). The evaluation also includes the RF post-processing that learns from LSTM and PEHM outputs (scheme HLR).

#### 3.2.1 PUB using a standalone model

Initially, we compared schemes H and L, which represent the conventional methods (without post-processing) for predicting streamflow in ungauged basins. The average prediction performance of the process-based model, PEHM, was the lowest among the seven schemes, with R^2^ = 0.6005 and NSE = 0.5238 (see Fig. 4). The average prediction performance of the data-driven model, LSTM, showed better results with R^2^ = 0.7365 and NSE = 0.6722. However, the average KGE for scheme L was 0.5237, lower than that for scheme H, which was 0.6525. Nonetheless, as mentioned earlier, these values represent satisfactory results above 0.5. Our findings are consistent with previous research results demonstrating the utility of both models for predicting streamflow in ungauged basins [3,26,34]. Since the types of the two models, PEHM and LSTM, used for streamflow prediction in ungauged basins in this study are different, there are fundamental differences in the approaches generally applied to predict ungauged basins. PEHM, which calculates daily hydrological elements such as evapotranspiration and infiltration through governing equations, and LSTM, which directly learns the relationship between long-term rainfall and streamflow, differ in calculation methods and training systems. Therefore, it should be noted that these differences may be reflected in our results.

#### 3.2.2 PUB using a hybrid model

The primary goal of this study is to examine whether post-processing through the combination of PBM and RF or DDM and RF can reduce the errors in the prediction results produced by each model.

The average performance of schemes H and HR does not differ substantially (see Fig. 4). The performance metrics (R^2^, NSE, and KGE) for scheme H are 0.6005, 0.5238, and 0.6525, respectively, which differ by ±0.03 from those of scheme HR (0.6241, 0.5496, and 0.6351). A notable observation is the differences in performance metrics between basins have decreased. Excluding outliers, the range of performance metrics for each basin decreased through post-processing. Compared to scheme H, the gap between the minimum and maximum values in scheme HR narrowed, particularly the minimum performance in R^2^ and NSE improved. This suggests that the results of the PBM, which showed significant performance variability across different ungauged basins, can achieve more stable performance through RF, which has learned information from multiple basins.

**Fig. 5:**
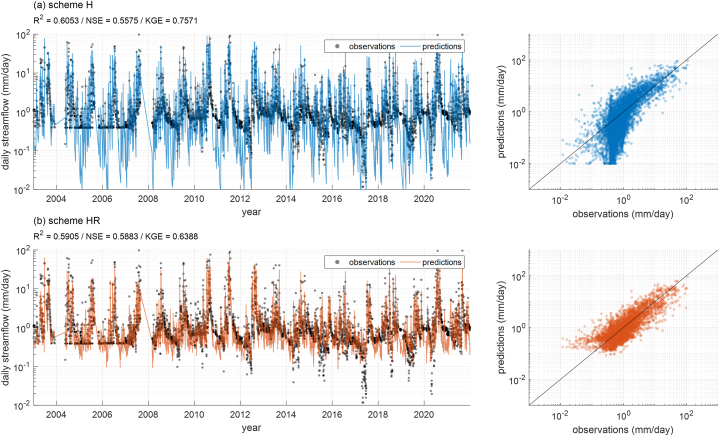
The results for Basin 15 from (a) scheme H and (b) scheme HR. In the left panel, the black points are the observed streamflow, and the blue and orange solid lines are the streamflow predicted by the model.

The left panel of Fig. 5 shows the observed and the predicted streamflow time series from scheme H and scheme HR for Basin 15, while the right panel displays a Q-Q plot of observed versus predicted values. Basin 15 represents a basin with average performance based on NSE, showing NSE = 0.5575 for scheme H and NSE = 0.5883 for scheme HR. The correction in prediction errors from scheme H through post-processing with RF can be visually confirmed in the results of scheme HR presented in Fig. 5b. Although there was no dramatic change in the average performance metrics, the results in Fig. 5 demonstrate that RF, which has learned the relationship between outputs and observations from various basins produced by PBM, can improve the errors in PBM predictions for ungauged basins and make the results more stable.

However, as observed in the hydrograph on the left side of Fig. 5, constant streamflow values were measured during the dry period between 2004 and 2007. This is considered to be an error caused by the limitations of the gauging equipment or unnatural stream behavior due to artificial reasons. Therefore, it cannot be concluded that the predictions of PEHM (scheme H) during this period are incorrect. Nevertheless, our results demonstrate that post-processing using RFs trained on observed data from other basins can reduce the discrepancies between predictions and observations inherent in PBMs. Conversely, this indirectly suggests that RFs, while not directly learning the errors of the target basin, might inadvertently capture similar observational errors from the gauged basins used for training, which could result in inaccurate outcomes.

**Fig. 6:**
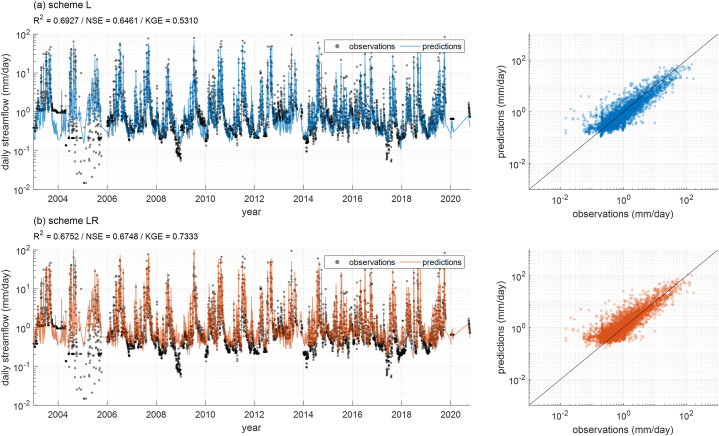
The results for Basin 12 from (a) scheme L and (b) scheme LR. In the left panel, the black points are the observed streamflow, and the blue and orange solid lines are the streamflow predicted by the model.

A notable difference between scheme L, which uses LSTM, and scheme LR, which undergoes a post-processing step with RF, is the change in KGE values. While R^2^ and NSE showed no significant change after post-processing with RF, the average KGE value significantly improved from 0.5237 to 0.7248 (see Fig. 4). Fig. 6 presents the time series and Q-Q plot of observed and predicted streamflow for Basin 12, representing the average results for schemes L and LR. However, differences between the time series of schemes L and LR were not visually identifiable, as presented in Fig. 6a and b.

**Fig. 7:**
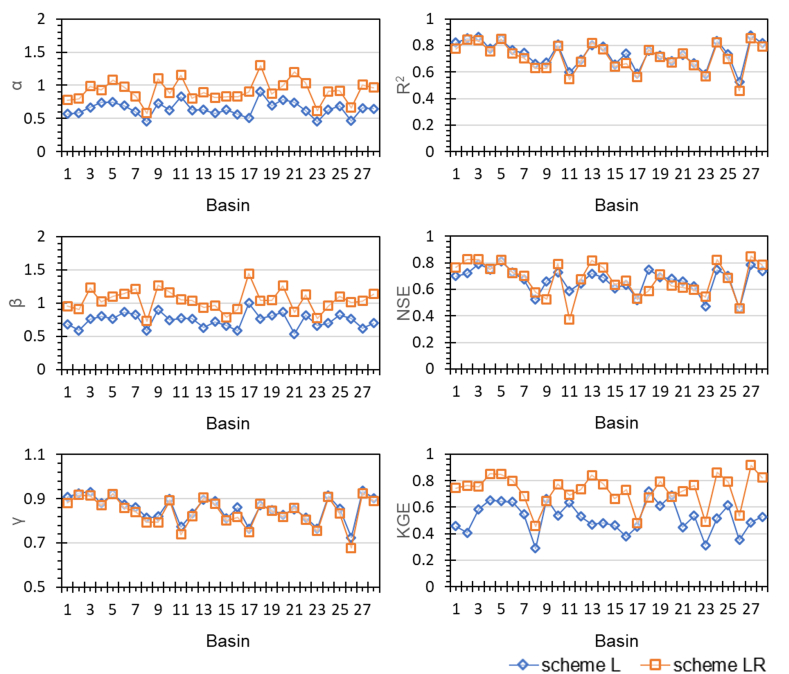
The ratio of means, ratio of standard deviations, correlation coefficients, and performance metrics for observed and predicted values by basins for schemes L and LR.

To clarify the reason for the large change in KGE due to post-processing, we separately identified the statistical factors for calculating KGE. The formula for calculating KGE (although not presented here) includes the ratio of the mean, the ratio of the standard deviation, and the correlation coefficient (denoted as α, β, and γ, respectively) between predicted and observed streamflows. The closer each component is to 1, the more similar the prediction is to the observation, indicating a higher KGE value. Fig. 7 displays the α, β, and γ for predictions by schemes L and LR, along with performance metrics for each basin. This shows that post-processing improved the mean (α) and standard deviation (β) of the predictions (making them closer to 1), but the correlation coefficient (γ) remained almost unchanged. Thus, post-processing with RF improved the mean and standard deviation of the predictions, except for a few cases. These improvements are not reflected in R^2^ and NSE but are captured in KGE. RF can make the mean and variance of predictions closer to those of the observations by averaging the results of each tree [58]. Therefore, our results demonstrate that post-processing with RF can improve the overall statistical characteristics (mean and standard deviation) of the predictions.

Ultimately, by learning from streamflow predictions from both types of models, scheme HLR can exhibit a compounded improvement effect. Compared to other schemes, the performance of streamflow predictions in ungauged basins by RF (scheme HLR), which learned from predictions by both PBM and DDM in gauged basins, shows relatively high R^2^ (0.7220) along with the highest average NSE and KGE (0.6912 and 0.7331) (see Fig. 4). This indicates that scheme HLR is the most competitive method.

#### 3.2.3 Comparison of predictions under hydrological conditions

**Fig. 8:**
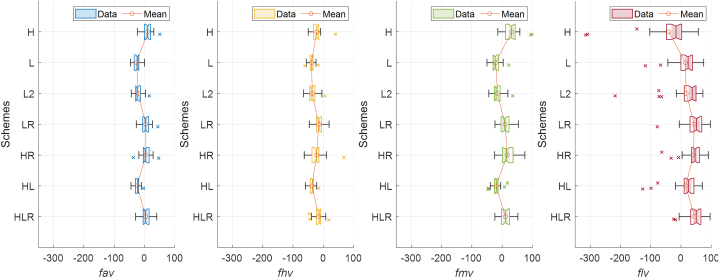
The Percent Bias (Pbias) between observed and predicted streamflows from each scheme. fav, fhv, fhv, and flv represent Pbias under overall, high-, medium-, and low-flow conditions, respectively. Each box shows the P-bias for the 28 ungauged basins, with the x-mark indicating outliers. The circle on the solid lines represents the average value.

The Flow Duration Curve (FDC), which describes the exceedance probability of streamflow, is one of the most important indicators of hydrological processes in a basin [59]. To quantitatively compare the FDCs across all basins, we compared their predictive performance by calculating Pbias under different hydrological conditions (see Section 2.5), described by exceedance probability intervals (see Fig. 8).

In Fig. 8, for P-bias of the overall period's flow (fav), post-processing improved the prediction performance to 5.0 % (scheme HR) and 4.2 % (scheme LR) from 10.6 % (scheme H) and −26.0 % (scheme L), respectively. Scheme HLR also showed improved performance with a P-bias of 4.6 %. The results varied depending on the scheme configuration when comparing the P-bias across different hydrological conditions. The combination with RF showed competitiveness in high-flow and medium-flow conditions. In high-flow conditions, scheme HLR had the lowest P-bias at −15.0 %; in medium-flow conditions, scheme LR had the lowest P-bias at 9.8 %. However, the combination with RF had a negative impact on low-flow conditions. Among the five schemes (H, L, HR, LR, and HLR), the results of LSTM without RF combination (scheme L) performed best in low-flow conditions, followed by scheme H. The schemes with post-processing (HR, LR, and HLR) resulted in larger biases than those without post-processing in low-flow conditions.

As previously mentioned, traditional Random Forest averages multiple decision tree results (ensembles). While this averaging helps prevent the model from overfitting, it can underestimate the variability of the response variable, impacting predictions for extreme values such as high and low flows [60,61]. However, in our study, although high-flow conditions are statistically more extreme (exceedance probability = 0.02) than low-flow (exceedance probability = 0.3), there is a statistical issue where errors have a greater impact on performance in low-flow conditions due to their smaller absolute values. For instance, an error of 0.01 mm may not significantly affect the prediction of a high flow of 100 mm, but it can relatively degrade the performance metrics in the case of a low flow of 0.1 mm. Moreover, as mentioned in Section 3.2.2, unnatural observations (observation errors) occurring in some low-flow periods also affect performance evaluation during low-flow conditions. Therefore, predictions combined with post-processing can secure better results in high- and medium-flow conditions. However, there are still questions regarding the prediction performance in low-flow conditions, necessitating further efforts to improve it.

### 3.3 Post-processing with RF or LSTM

In section 3.2.2, we observed that while post-processing with RF does not significantly impact the improvement of performance metrics for the predictions by PEHM. Consequently, we sought to further explore the utility of using LSTM as a post-processor in comparison to RF. Compared to the results of post-processing the predictions from scheme H with RF, the results post-processed with LSTM showed a relative improvement in average R^2^ and NSE, while KGE experienced a relative decrease (see Fig. 4).

**Fig. 9:**
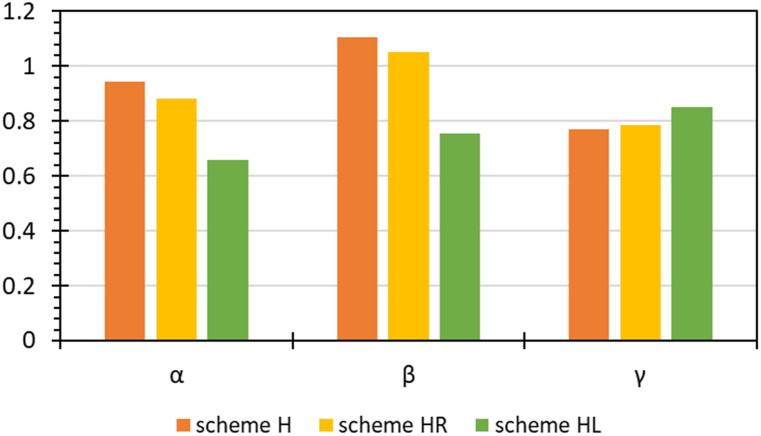
Basin averages for statistical components for schemes H, HR, and HL. α represents the ratio of means between predicted and observed streamflows, β represents the ratio of standard deviations, and γ represents the correlation coefficient. The closer each component is to 1, the more similar the predictions are to the observations.

Fig. 9 shows the basin averages for the ratio of means (α), the ratio of standard deviations (β), and the correlation coefficient (γ) between the observations and predictions for schemes H, HR, and HL. As confirmed in Section 3.2.2, post-processing with RF does not significantly impact the improvement of statistical components for the results predicted by PEHM. In contrast, post-processing with LSTM improved the correlation between predictions and observations, improving the R^2^ and NSE metrics. This success of LSTM can be attributed to its ability to effectively learn complex patterns and long-term dependencies in time series data. Particularly, LSTM's effectiveness in learning time-varying hydrological processes can enhance the correlation between predictions and observations when used as a post-processor. However, it should be noted that the accuracy of predictions, as indicated by the errors in mean and standard deviation, worsened, which is a limitation reflected in the decrease in KGE values. This suggests that a comprehensive evaluation of the model's performance should consider various metrics when selecting a post-processing technique.

P-bias presents another difference between the two post-processing models. As shown in Fig. 8, compared to scheme H, scheme HR shows strength in improving fav and fmv, but there is no significant change in P-bias for high- and low-flow conditions (fhv and flv). On the other hand, scheme HL does not show improvement in high-flow conditions but generally improves in low- and medium-flow conditions. These results highlight the differences between RF, which has advantages in improving the mean, and LSTM, which excels in capturing long-term dependencies. One of the reasons LSTM, which learns the long-term behavior of water, may better describe medium- and low-flow conditions compared to RF, which relies solely on immediate information, is due to this characteristic.

Accurate prediction of low flows is crucial for water resource plans to maintain healthy watershed ecosystems [62,63]. While hydrological models generally simulate medium and high flows reasonably well, accurately predicting low flows remains a significant challenge [64]. Our results indicate that post-processing with RF generally provides relatively better prediction performance in most situations, but it has limitations in improving low-flow predictions (see Section 3.2.3). In situations focused on low-flow predictions, such as drought analysis, post-processing based on LSTM might be a better option.

### 3.4 Use of topographic information (scheme L, L2, and LR)

In the traditional hydrological model's regionalization approach, the selection of a donor basin plays a crucial role in determining the prediction performance for the target basin (the ungauged basin). Typically, basins with similar hydrological processes are chosen as donor basins, with a preference often given to adjacent basins that share similar topographic conditions [1]. While PBMs incorporate various topographic information (such as area and impervious surface ratio) in the model construction process, general DDMs learn only the relationship between precipitation (P) and streamflow (Q). In our method of predicting streamflow in ungauged basins by learning from gauged basin information, we aimed to assess the extent to which the inclusion of topographic information impacts DDM performance. In other words, the comparison of schemes L, L2, and LR can show how the prediction performance changes depending on whether and how topographic information is learned. Scheme L does not include topographic information, scheme L2 adds topographic information during the recurrent neural network phase, and scheme LR incorporates topographic information during the post-processing phase. Compared to scheme L, which learned the relationship between input (precipitation, meteorological data) and target (streamflow) in gauged basins, scheme L2, which added topographic information as input, did not significantly affect the performance of streamflow prediction in ungauged basins (see Fig. 4). Adding topographic information to the training of the LSTM changed the R^2^, NSE, and KGE by −0.0278, +0.0285, and −0.0222 on average, respectively. The performance improvement in scheme LR is inferred to be due to the post-processing step rather than the addition of topographical information. Choi et al. [65] reported that including topographical information might not play a significant role in the model's performance in their evaluation of LSTM's regionalization performance using 13 basins in Korea. Similarly, Won et al. [26] found that the additional use of topographical data in a hybrid strategy combining RNN and DT-based algorithms did not dramatically improve the prediction performance for ungauged basins, and the importance of the added topographical data within the model's prediction process was not high. However, it should be noted that this study only utilized the same limited type of hydrogeomorphical information the was used in the PEHM. Utilizing more comprehensive information could potentially yield different results.

Since our main objective was not to assess the utility of topographical information, we did not conduct further detailed analysis. However, Liu et al. [66] recently integrated 27 static basin attributes into a hybrid model combining the Danish National Water Resources Model (DKM) and LSTM, and these attributes contributed to improving the accuracy of streamflow predictions. Similarly, Tifru et al. [67] mentioned that basin attributes (such as land use, topography, and soil composition) are crucial factors in streamflow prediction when using hybrid models. As the hydrological and topographical information of a basin remains valuable for distinguishing features between basins that exhibit different hydrological processes, further research is needed to leverage this information for better predictions, such as by defining topographical information as environmental variables using entity-aware LSTM.

### 3.5 Role and limitations of post-processing techniques in PUB

PUB is an important challenge for water resource management and environmental protection in watersheds with scarce or no data. Various modeling approaches using PBMs and DDMs have been used to make these predictions, each with its own strengths and weaknesses. While hybrid models incorporating DDMs have been applied in research aiming to predict streamflow in gauged basins [39,68], most studies focusing on ungauged basin streamflow prediction have utilized either PBM or DDM alone [25,[33], [34], [35], [36], [37]]. In this study, we initially predicted streamflow behavior using PBM (i.e., PEHM) and/or DDM (i.e., LSTM) networks. Subsequently, we employed Random Forest (RF) linked with meteorological data and basin characteristics to make the final streamflow predictions. Attempting frameworks that sequentially combine these two steps and analyze their differences to predict streamflow in ungauged basins represents the most significant innovation of this research. Our results show that post-processing with RF has a meaningful impact on improving the prediction of ungauged basins. In particular, the best prediction results are obtained by simultaneously post-processing PEHM and LSTM predictions with RF. Our experiments demonstrate that post-processing techniques play a crucial role in enhancing the performance of hydrological prediction models. These techniques can contribute to reducing prediction errors and increasing accuracy. However, the effectiveness of post-processing techniques can vary depending on the models used, the hydrological conditions applied, and the characteristics of the data. In this study, post-processing with RF, which calculates the average of an ensemble, was helpful in improving the primary prediction results but did not aid in improving the prediction performance for low-flow (see Section 3.2.3). On the other hand, LSTM, which learns long-term dependencies, was specialized in addressing errors in low-flow predictions (see Section 3.3). Thus, each model can help solve different aspects of errors that may occur in predictions. This implies that for the practical application of post-processing techniques, it is essential to accurately understand the error patterns in model outputs and select the appropriate post-processing technique to correct these errors. Therefore, exploring the application of various models as post-processing processors, considering the basic assumptions of the model and the characteristics of the predictions aimed, would also be a valuable research topic.

Additionally, DDMs perform well when a large amount of data is available [53]. A vast training dataset helps the network learn more general and abstract patterns in the input-output relationship. In this study, we confirmed that integrating information from many gauged basins for prediction or post-processing is beneficial for PUB. However, this study is limited to experiments conducted in 28 basins within Korea, which generally possess semi-humid hydrological characteristics. Given the fact that hydrological processes are different in arid and humid basins, further research should cover more basins to determine which approach is more beneficial: integrating all data regardless of hydrological characteristics or categorizing basins characteristics and then pooling basins with similar characteristics.

## 4 Conclusion

This study investigated the applicability of DDMs as post-processing processors to reduce the prediction errors of streamflow in ungauged basins using PBM and DDM. The results suggest that applying post-processing techniques using RF can enhance the accuracy of streamflow predictions in ungauged basins derived from PBM and/or DDM. This combination contributes to reducing the model's prediction errors, thereby improving the prediction accuracy and reliability. The main findings of this study are as follows.

(1) Both the single and hybrid models constructed using PBM and DDMs in this study can satisfactorily predict streamflow in ungauged basins;
(2) Post-processing with RF can help improve the statistics of LSTM predictions;
(3) The method of post-processing the streamflow predictions from both PEHM and LSTM simultaneously with RF yielded the highest prediction performance for streamflow in ungauged basins;
(4) However, post-processing with RF showed limitations in improving low-flow predictions; and
(5) Separately, post-processing with LSTM demonstrated a specialized advantage in improving low-flow predictions.

This study demonstrates the potential value of various post-processing approaches in improving streamflow predictions in ungauged basins. It also emphasizes that the effect on prediction improvement varies depending on the unique characteristics of the chosen post-processing model. However, additional validation is needed across various basins exhibiting different hydrological conditions. In developing and applying post-processing techniques, continually exploring methods to reduce prediction errors through additional applications across various models and basins is expected to enhance hydrological prediction models' reliability and accuracy.

## CRediT authorship contribution statement

**Jeonghyeon Choi:** Writing – original draft, Visualization, Validation, Software, Methodology, Investigation, Formal analysis, Data curation, Conceptualization. **Sangdan Kim:** Writing – review & editing, Validation, Supervision, Project administration, Methodology, Conceptualization.

## Ethics approval and consent to participate

Not applicable.

## Consent for publication

All authors have read and approved the final manuscript and consent to its publication in Heliyon.

## Data availability statement

Data will be made available on request.

## Declaration of competing interest

The authors declare that they have no known competing financial interests or personal relationships that could have appeared to influence the work reported in this paper.
